# Synthesis and crystal structure of *anti*-10-(4-cyano­phen­yl)-10,11,22,23-tetra­hydro-9*H*,21*H*-5,8:15,12-bis­(metheno)[1,5,11]tri­aza­cyclo­hexadecino[1,16-*a*:5,6-*a*′]di­indole di­chloro­methane monosolvate

**DOI:** 10.1107/S2056989024011782

**Published:** 2025-01-01

**Authors:** Koji Kubono, Keita Tani, Yukiyasu Kashiwagi

**Affiliations:** a Osaka Kyoiku University, 4-698-1 Asahigaoka Kashiwara Osaka 582-8582 Japan; b Osaka Research Institute of Industrial Science and Technology, 1-6-50 Morinomiya, Joto-ku, Osaka 536-8553, Japan; University of Hyogo, Japan

**Keywords:** crystal structure, carbazole, C—H⋯π inter­actions

## Abstract

The asymmetric unit of the title compound is composed of one host mol­ecule, *anti*-4-(1^9^*H*,5^9^*H*-3-aza-1,5(3,9)-dicarbazola­cyclo­octa­phane-3-yl)benzo­nitrile and one di­chloro­methane solvate mol­ecule, C_36_H_28_N_4_·CH_2_Cl_2_. The host mol­ecule possesses a planar chirality but crystallizes as a racemate in the space group *P*2_1_/*c*. It adopts an *anti*-configuration, in which two carbazole rings are partially overlapped with a parallel orientation. The two carbazole ring systems are slightly bent, and the C atoms at the 3- and 1-positions show the largest deviations from the mean planes. The dihedral angle between two carbazole rings is 9.42 (3)°, forming an intra­molecular parallel π–π inter­action [*Cg*⋯*Cg* = 3.2755 (9) Å]. In the crystal, the mol­ecules are linked *via* host–host and host–guest C—H⋯π inter­actions, forming chain structures along the *a*-axis direction. The mol­ecules are linked into a ribbon structure along the *c-*axis direction by further C—H⋯π inter­actions. As a result, The mol­ecules are cross-linked by C—H⋯π inter­actions into a three-dimensional network.

## Abstract 2

The title compound consists of one *anti*-4-(1^9^*H*,5^9^*H*-3-aza-1,5(3,9)-dicarbazola­cyclo­octa­phane-3-yl)benzo­nitrile (host mol­ecule) and one di­chloro­methane solvate mol­ecule. The host mol­ecule adopts an *anti* configuration, in which two carbazole rings are partially overlapped, forming an intra­molecular π–π inter­action. In the crystal, the mol­ecules are cross-linked *via* inter­molecular host–host and host–guest C—H⋯π inter­actions, forming a three-dimensional network.

## 1. Chemical context

Many carbazole derivatives emit blue fluorescence in good quantum yields, and have been used in the development of organic light-emitting diodes (Chen *et al.*, 2021 ▸). The carbazole chromophore, which shows donor character, has been employed as a hole-transporting material in organic solar cells (Konidena *et al.*, 2022 ▸). As for the structure of the excimer in carbazole chromophore, partially overlapped (PO) and fully overlapped (FO) excimers were proposed (Sakai *et al.*, 1996 ▸). To investigate the structure and photophysical properties of the carbazole excimer, our group has synthesized various carbazolophanes (CZPs), which are cyclo­phanes composed of two carbazole rings (Tani *et al.*, 1996 ▸; Benten *et al.*, 2005 ▸). The framework of [3.3](3,9)- and [3.4](3,9)-CZPs, where (3,9) describes the bridging position of carbazole ring, and [*m.n*] denotes the number of bridging lengths between the 3- and 9-positions, are rigid enough to isolate both PO (*anti*) and FO (*syn*) isomers at room temperature. For [3.3](3,9)-CZPs, flipping of the carbazole ring between *syn* and *anti* CZPs does not occur at room temperature, therefore *anti* CZPs with planar chirality were successfully separated as enanti­omers (Tani *et al.*, 2020 ▸). Intriguingly, the fluorescence spectrum of cyanamide-bridged [3.3](3,9)-CZP was assigned to be excimeric emission, while monomer-like emission was observed in [3.4](3,9)-CZP (Tani *et al.*, 2007 ▸). This result indicates that excimer formation in the carbazole chromophore is extremely susceptible to the geometry of the two carbazole rings in close proximity. As the [3.3](3,9)-aza-bridged CZPs synthesized so far were *N*-sulfonamide-bridged CZP (Tani *et al.*, 2020 ▸), *N*-cyanamide-bridged CZP (Tani *et al.*, 2001 ▸), and *N*-*n*-butyl­amine-bridged CZP (Kubono *et al.*, 2022 ▸), we plan to synthesize more basic aromatic amine-bridged CZP, that is, an aniline derivative-bridged one, which is a potential candidate for systematic elucidation of the excimer formation in the carbazole chromophore. 4-Cyano­aniline was chosen to begin this research since it is treated as the insertion of phenyl­ene moiety into cyanamide and the effect of the aromatic ring can be evaluated. The cyclization reaction between 4-cyano­aniline and 9,9-(1,3-propanedi­yl)-bis­[3-(bromo­meth­yl)-9*H*-carb­azole] gave the title com­pound, *anti*-10-(4-cyano­phen­yl)-10,11,22,23-tetra­hydro-9*H*,21*H*-5,8:15,12-bis­(metheno)[1,5,11]tri­aza­cyclo­hexa­decino[1,16-*a*:5,6*a*′]di­indole[cyclo­phane nomenclature: *anti*-4-(1^9^*H*,5^9^*H*-3-aza-1,5(3,9)-dicarb­azola­cyclo­octaphane-3-yl)ben­zo­nitrile] di­chloro­methane solvate. Herein we report on the synthesis and crystal structure of it.
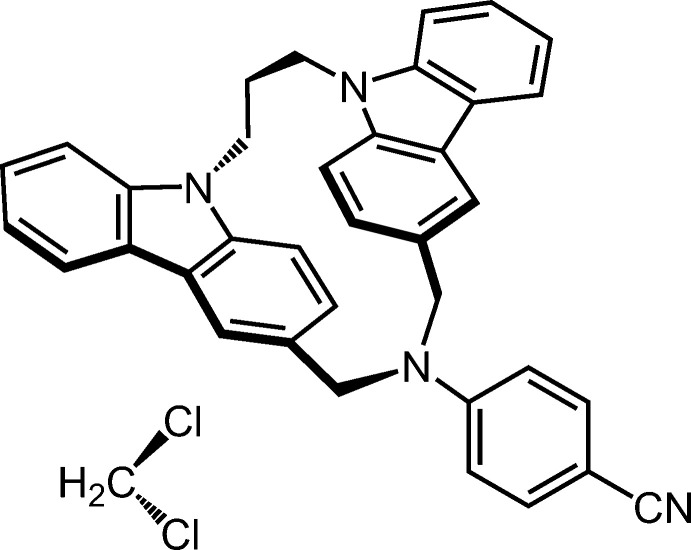


## 2. Structural commentary

**Figure 1:**
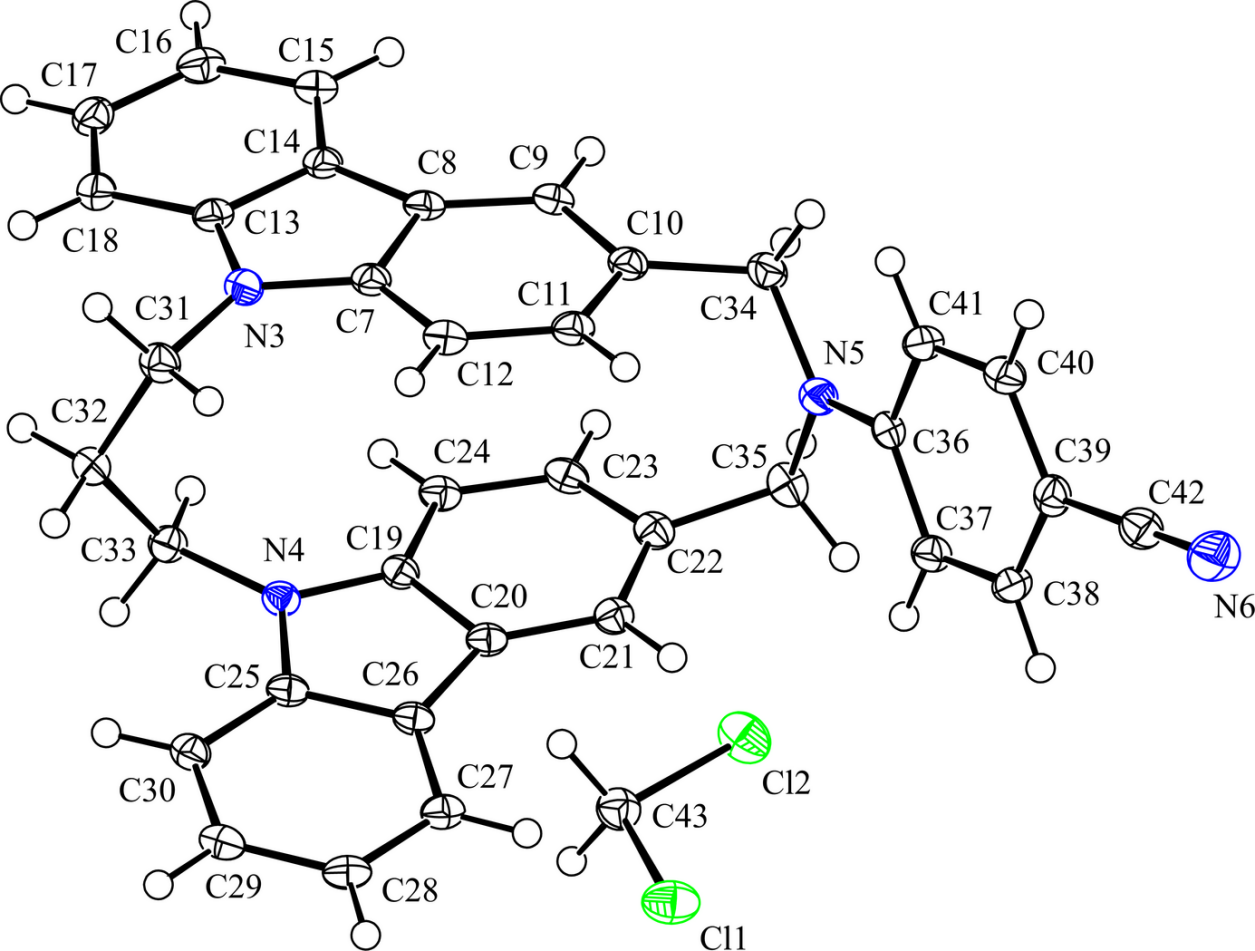
The mol­ecular structure of the title compound, with the atom labelling. Displacement ellipsoids are drawn at the 50% probability level. H atoms are represented by spheres of arbitrary radius.

The asymmetric unit of the title compound is composed of one host mol­ecule and one di­chloro­methane solvate mol­ecule (Fig. 1 ▸). The host mol­ecule of the title compound possesses a planar chirality but crystallizes as a racemate in the centrosymmetric space group *P*2_1_/*c*. The host mol­ecule adopts an *anti*-configuration with parallel orientation, thus it is classified into a PO-CZP structure. The carbazole ring systems are slightly bent, with r.m.s. deviations of 0.066 (1) and 0.078 (1) Å, respectively, for the N3/C7–C18 ring and N4/C19–C30 ring systems. In two carbazole fragments, the C atoms at the 3- and 1-positions of carbazole ring show the largest deviations from mean planes [−0.1174 (15) Å for C10 (3-position) and −0.1219 (14) Å for C24 (1-position)]. The dihedral angle between two carbazole fragments is 9.42 (3)°, providing an intra­molecular π–π inter­action [*Cg*3⋯*Cg*4 = 3.2755 (9) Å; *Cg*3 and *Cg*4 are the centroids of rings C7–C12 and C19–C24, respectively]. In comparison, the dihedral angle between the two carbazole rings and *Cg*⋯*Cg* distances in the crystal structures of related compounds are 5.19 (3)° and 3.2514 (8) Å for [3.3](3,9)-*N*-*n*-butyl­amine-bridged PO-CZP (XEBDAN; Kubono *et al.*, 2022 ▸) and 9.65 (5)° and 3.2296 (12) Å for [3.3](3,9)-*N*-(*R*)-phenethyl­amine-bridged (*S*_p_)-PO-CZP (YOLRAW; Tani *et al.*, 2023 ▸), which are close to those of the host mol­ecule in the title compound. The bond angle C34—N5—C35 is 114.81 (11)° in the title compound, similar to those of the related compounds [114.56 (11)° for XEBDAN; 113.97 (16)° for YOLRAW]. For the related compound with a cyano­aniline moiety, 4-(di­benzyl­amino)­benzo­nitrile, the average C(methyl­ene)—N—C bond angle in the two independent mol­ecules in the crystal (IYAXOY; Luo *et al.*, 2021 ▸) is 115.97 (12)°. The N5 atom is located 0.0067 (11) Å above the mean plane of the three bounded carbon atoms (C34/C35/C36) in the tertiary amino group. The N5 atom has highly *sp*^2^ orbital character because this bridged amine contains an aromatic moiety, whose nitrile group in the 4-position is an electron-withdrawing one.

## 3. Supra­molecular features

**Figure 2:**
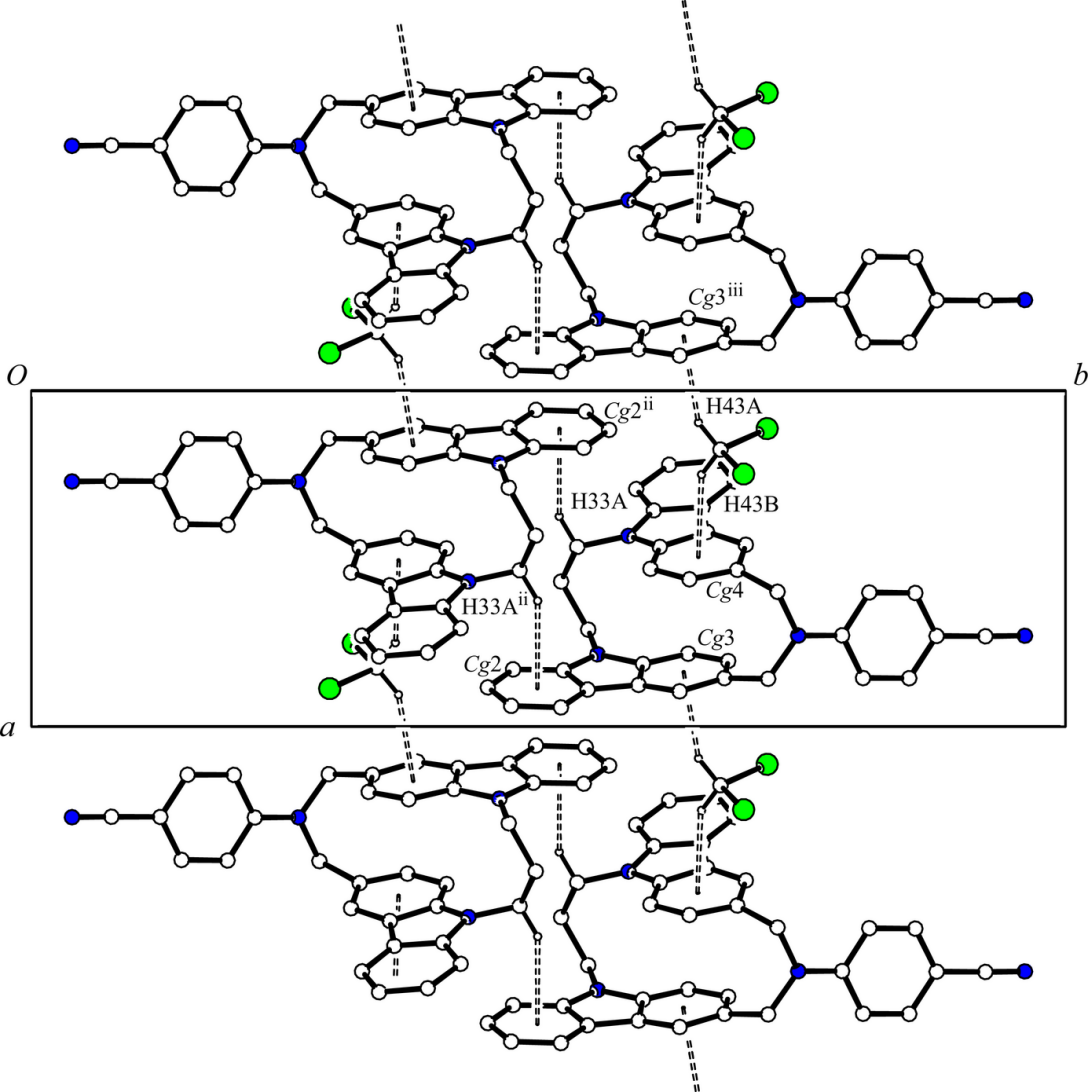
A packing diagram of the title compound viewed along the *c* axis, showing the chain structure. The C—H⋯π inter­actions are shown as double dashed lines. H atoms not involved in the inter­actions have been omitted for clarity. [Symmetry codes: (ii) 1 − *x*, 1 − *y*, 1 − *z*; (iii) *x -* 1, *y*, *z*.]

**Figure 3:**
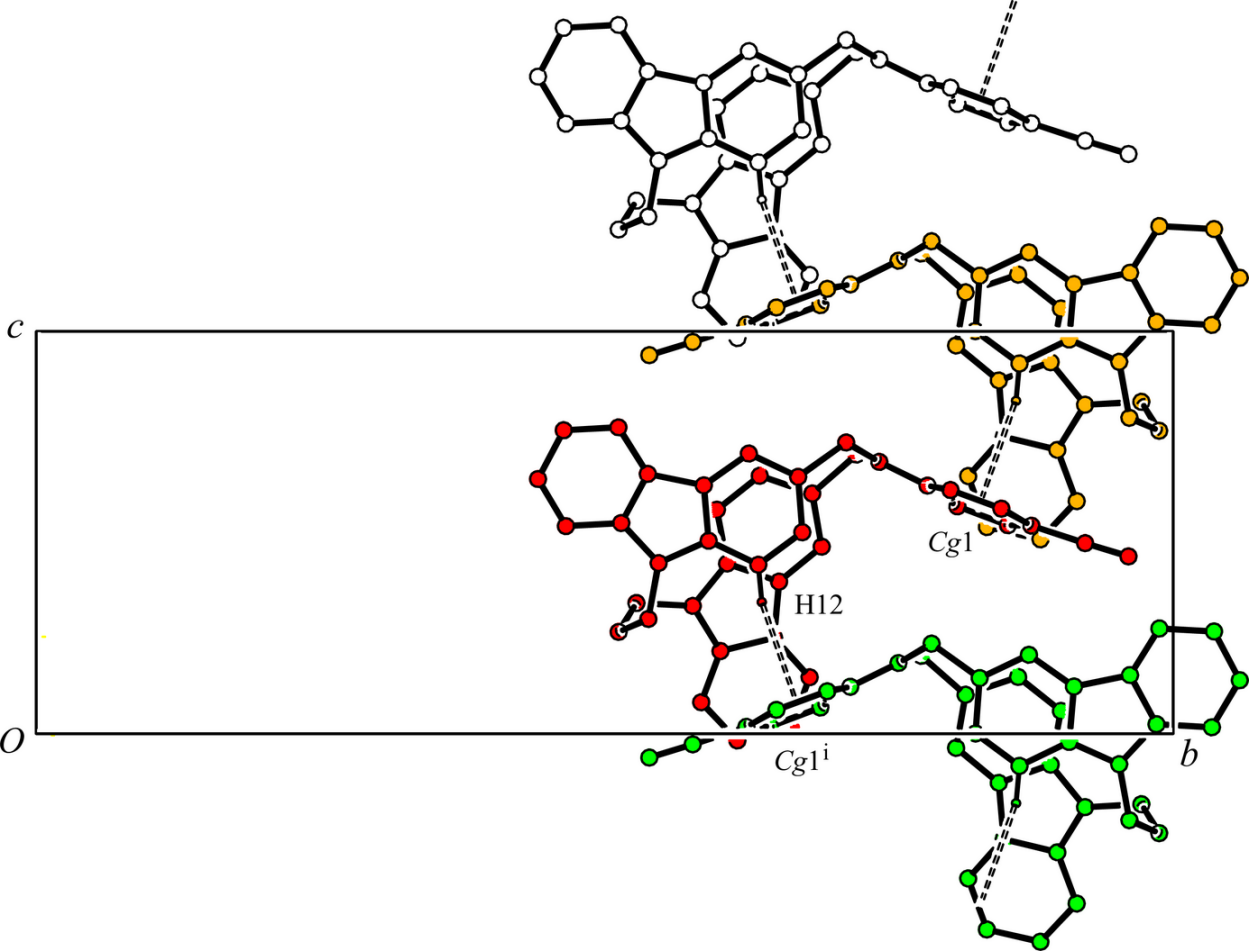
A packing diagram of the title compound viewed along the *a* axis, showing the ribbon structure. C—H⋯π inter­actions are shown as double dashed lines. H atoms not involved in the inter­actions have been omitted for clarity. [symmetry code: (i) *x*, 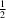 − *y*, *z* − 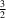.]

In the crystal, two mol­ecules are associated through a pair of inter­molecular C—H⋯π inter­actions [C33—H33*A*⋯*Cg*2^ii^; H33*A*⋯*Cg*2^ii^ = 2.91 Å; C33⋯*Cg*2^ii^ = 3.6402 (17)Å; *Cg*2 is the centroid of the C13–C18 ring, symmetry code: (ii) 1 − *x*, 1 − *y*, 1 − *z*] (Table 1 ▸), forming a centrosymmetric dimer. The dimers and solvate di­chloro­methane mol­ecules are linked by two other C—H⋯π inter­actions [C43—H43*A*⋯*Cg*3^iii^; H43*A*⋯*Cg*3^iii^ = 2.56 Å; C43⋯*Cg*3^iii^ = 3.4632 (17) Å, and C43—H43*B*⋯*Cg*4; H43*B*⋯*Cg*4 = 2.54 Å; C43⋯*Cg*4 = 3.4277 (17) Å; *Cg*3 and *Cg*4 are the centroids of the C7–C12 and C19–C24 rings, respectively; symmetry code: (iii) *x -* 1, *y*, *z*] (Table 1 ▸), forming chain structures along the *a-*axis direction (Fig. 2 ▸). In addition, the host mol­ecules linked by another C—H⋯π inter­action [C12⋯H12⋯*Cg*1^i^; H12⋯*Cg*1^i^ = 2.93 Å; C12⋯*Cg*1 = 3.8374 (15) Å; *Cg*1 is the centroid of the C36–C41 ring; symmetry code: (i) *x*, 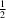 − *y*, *z* − 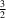] (Table 1 ▸), forming a ribbon structure along the *c-*axis direction (Fig. 3 ▸). As a result, the mol­ecules are cross-linked *via* C—H⋯π inter­actions into a three-dimensional network.

## 4. Database survey

A search of the Cambridge Structural Database (CSD, Version 2024.1.0, update of March 2024; Groom *et al.*, 2016 ▸) using *ConQuest* (Bruno *et al.*, 2002 ▸) for compounds containing carbazole skeleton gave 6572 hits, and for those containing two 3,9-di­methyl­enecarbazole fragments gave 573 hits. Among those, the [3.3](3,9)-CZP skeleton gave four hits. Of these four compounds, three structures are PO-carbazolophanes with the same skeleton as the title compound, [3.3](3,9)-*N*-sulfonamide-bridged PO-CZP (YUKYEL; Tani *et al.*, 2020 ▸), [3.3](3,9)-*N*-cyanamide-bridged PO-CZP (BACKOG; Tani *et al.*, 2001 ▸), and [3.3](3,9)-*N*-*n*-butyl­amine-bridged PO-CZP (XEBDAN; Kubono *et al.*, 2022 ▸). One structure is [3.3](3,9)-*N*-cyanamide-bridged fully overlapped (FO)-CZP, *syn*-3-cyano-3-aza-1(9,3),3(3,9)-dicarbazola­cyclo­octa­phane benzene clathrate (BACKIA; Tani *et al.*, 2001 ▸). In addition to these, we have recently reported the structures of newly chiral [3.3](3,9)-*N*-(*R*)-phenethyl­amine-bridged (*S*_p_)-PO-CZP (YOLRAW; Tani *et al.*, 2023 ▸).

## 5. Synthesis and crystallization

A solution of 9,9′-(1,3-propanedi­yl)bis­[3-(bromo­meth­yl)-9*H-*carbazole] (370 mg, 0.66 mmol; Tani *et al.*, 2001 ▸) in di­chloro­methane (200 mL) was added to a 500 mL flask, which contained a mixture of tetra­butyl­ammonium iodide (85.0 mg, 0.23 mmol) and 4-cyano­aniline (82 mg, 0.69 mmol) in di­chloro­methane ­(150 mL) and sodium hydroxide (1.16 g, 29 mmol) in water (10 mL). Then, the flask was filled with argon and was stirred at room temperature for 3 d. The reaction mixture was washed with water, then the organic layer was washed with sat. aq. NaCl, and dried over anhydrous sodium sulfate. The solvent was removed under reduced pressure, and the residue was purified by silica gel chromatography (Wako-gel C-200, 10 g). Elution from benzene gave a white solid (21.2 mg, 0.041 mmol, 6%). It was recrystallized from di­chloro­methane:ethanol (1:3) to give a colourless crystal of the title compound suitable for X-ray diffraction. Melting point (decomposition): 561–563 K. ^1^H NMR (CDCl_3_, 400 MHz): *δ* = 2.88–2.97 (*m*, 2H), 3.71–3.81 (*m*, 2H), 4.12–4.20 (*m*, 2H), 4.79, 4.99 (*ABq*, *J* = 15.6 Hz, 4H), 5.35 (*d*, *J* = 8.4 Hz, 2H), 6.19 (*d*, *J* = 8.8 Hz, 2H), 7.23–7.33 (*m*, 4H), 7.48–7.55 (*m*, 4H), 7.62 (*d*, *J* = 8.4 Hz, 2H), 7.74 (*s*, 2H), 8.08 (*d*, *J* = 7.2 Hz, 2H).

## 6. Refinement

Crystal data, data collection and structure refinement details are summarized in Table 2 ▸. The hy­droxy H atoms were located in a difference-Fourier map and freely refined. The C-bound H atoms were placed in geometrically calculated positions (C—H = 0.95–0.99 Å) and refined as part of a riding model with *U*_iso_(H) = 1.2 *U*_eq_ (C).

## Supplementary Material

Crystal structure: contains datablock(s) I. DOI: 10.1107/S2056989024011782/ox2009sup1.cif

Structure factors: contains datablock(s) I. DOI: 10.1107/S2056989024011782/ox2009Isup2.hkl

CCDC references: 2407492, 2407492

Additional supporting information:  crystallographic information; 3D view; checkCIF report

## Figures and Tables

**Table 1:** Hydrogen-bond geometry (Å, °) *Cg*1, *Cg*2, *Cg*3 and *Cg*4 are the centroids of rings C36–C41, C13–C18, C7–C12 and C19–C24, respectively.

*D*—H⋯*A*	*D*—H	H⋯*A*	*D*⋯*A*	*D*—H⋯*A*
C12—H12⋯*Cg*1^i^	0.95	2.93	3.8374 (15)	159
C33—H33*A*⋯*Cg*2^ii^	0.99	2.91	3.6402 (17)	131
C43—H43*A*⋯*Cg*3^iii^	0.99	2.56	3.4632 (17)	151
C43—H43*B*⋯*Cg*4	0.99	2.54	3.4277 (17)	149

Symmetry codes: (i) 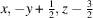; (ii) 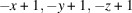; (iii) 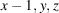.

**Table 2:** Experimental details

Crystal data	
Chemical formula	C_36_H_28_N_4_·CH_2_Cl_2_
*M*_r_	601.55
Crystal system, space group	Monoclinic, *P*2_1_/*c*
Temperature (K)	100
*a*, *b*, *c* (Å)	9.8527 (1), 28.7160 (3), 10.7754 (1)
β (°)	109.085 (2)
*V* (Å^3^)	2881.11 (6)
*Z*	4
Radiation type	Cu *K*α
μ (mm^−1^)	2.29
Crystal size (mm)	0.29 × 0.26 × 0.22

Data collection	
Diffractometer	XtaLAB Synergy, Dualflex, HyPix
Absorption correction	Multi-scan (*CrysAlis PRO*; Rigaku OD, 2023 ▸)
*T*_min_, *T*_max_	0.726, 1.000
No. of measured, independent and observed [*I* > 2σ(*I*)] reflections	20903, 5741, 5247
*R*_int_	0.032
(sin θ/λ)_max_ (Å^−1^)	0.632

Refinement	
*R*[*F*^2^ > 2σ(*F*^2^)], *wR*(*F*^2^), *S*	0.034, 0.090, 1.05
No. of reflections	5741
No. of parameters	388
H-atom treatment	H-atom parameters constrained
Δρ_max_, Δρ_min_ (e Å^−3^)	0.25, −0.37

Computer programs: *CrysAlis PRO* (Rigaku OD, 2023 ▸), *SHELXT2018/2* (Sheldrick, 2015*a* ▸), *SHELXL2018/3* (Sheldrick, 2015*b* ▸), *PLATON* (Spek, 2020 ▸) and *OLEX2* (Dolomanov *et al.*, 2009 ▸).

## supplementary crystallographic information

### 10-(4-Cyanophenyl)-10,11,22,23-tetrahydro-9H,21H-5,8:15,12-bis(metheno)[1,5,11]triazacyclohexadecino[1,16-a:5,6-a']diindole dichloromethane monosolvate Crystal data

**Table:**

C_36_H_28_N_4_·CH_2_Cl_2_	*F*(000) = 1256
*M**_r_* = 601.55	*D*_x_ = 1.387 Mg m^−^^3^
Monoclinic, *P*2_1_/*c*	Cu *K*α radiation, λ = 1.54184 Å
*a* = 9.8527 (1) Å	Cell parameters from 14622 reflections
*b* = 28.7160 (3) Å	θ = 3.1–76.9°
*c* = 10.7754 (1) Å	µ = 2.29 mm^−^^1^
β = 109.085 (2)°	*T* = 100 K
*V* = 2881.11 (6) Å^3^	Block, colourless
*Z* = 4	0.29 × 0.26 × 0.22 mm

### 10-(4-Cyanophenyl)-10,11,22,23-tetrahydro-9H,21H-5,8:15,12-bis(metheno)[1,5,11]triazacyclohexadecino[1,16-a:5,6-a']diindole dichloromethane monosolvate Data collection

**Table:**

XtaLAB Synergy, Dualflex, HyPix diffractometer	5741 independent reflections
Radiation source: micro-focus sealed X-ray tube, PhotonJet (Cu) X-ray Source	5247 reflections with *I* > 2σ(*I*)
Mirror monochromator	*R*_int_ = 0.032
Detector resolution: 10.0000 pixels mm^-1^	θ_max_ = 77.1°, θ_min_ = 3.1°
ω scans	*h* = −12→12
Absorption correction: multi-scan (CrysAlisPro; Rigaku OD, 2023)	*k* = −34→35
*T*_min_ = 0.726, *T*_max_ = 1.000	*l* = −13→12
20903 measured reflections	

### 10-(4-Cyanophenyl)-10,11,22,23-tetrahydro-9H,21H-5,8:15,12-bis(metheno)[1,5,11]triazacyclohexadecino[1,16-a:5,6-a']diindole dichloromethane monosolvate Refinement

**Table:**

Refinement on *F*^2^	0 restraints
Least-squares matrix: full	Hydrogen site location: inferred from neighbouring sites
*R*[*F*^2^ > 2σ(*F*^2^)] = 0.034	H-atom parameters constrained
*wR*(*F*^2^) = 0.090	*w* = 1/[σ^2^(*F*_o_^2^) + (0.042*P*)^2^ + 1.3432*P*] where *P* = (*F*_o_^2^ + 2*F*_c_^2^)/3
*S* = 1.05	(Δ/σ)_max_ = 0.001
5741 reflections	Δρ_max_ = 0.25 e Å^−^^3^
388 parameters	Δρ_min_ = −0.37 e Å^−^^3^

### 10-(4-Cyanophenyl)-10,11,22,23-tetrahydro-9H,21H-5,8:15,12-bis(metheno)[1,5,11]triazacyclohexadecino[1,16-a:5,6-a']diindole dichloromethane monosolvate Special details

**Table:**

Geometry. All esds (except the esd in the dihedral angle between two l.s. planes) are estimated using the full covariance matrix. The cell esds are taken into account individually in the estimation of esds in distances, angles and torsion angles; correlations between esds in cell parameters are only used when they are defined by crystal symmetry. An approximate (isotropic) treatment of cell esds is used for estimating esds involving l.s. planes.

### 10-(4-Cyanophenyl)-10,11,22,23-tetrahydro-9H,21H-5,8:15,12-bis(metheno)[1,5,11]triazacyclohexadecino[1,16-a:5,6-a']diindole dichloromethane monosolvate Fractional atomic coordinates and isotropic or equivalent isotropic displacement parameters (Å^2^)

**Table:**

	*x*	*y*	*z*	*U*_iso_*/*U*_eq_	
Cl1	0.11268 (4)	0.71150 (2)	0.44555 (4)	0.02877 (10)	
Cl2	0.24825 (4)	0.68876 (2)	0.72262 (3)	0.02901 (10)	
N4	0.43170 (12)	0.57748 (4)	0.31773 (11)	0.0156 (2)	
N3	0.78473 (12)	0.54742 (4)	0.42453 (11)	0.0174 (2)	
N5	0.72941 (13)	0.74158 (4)	0.67578 (11)	0.0185 (2)	
N6	0.72965 (16)	0.96072 (5)	0.44091 (14)	0.0309 (3)	
C25	0.35580 (14)	0.60184 (5)	0.20569 (13)	0.0160 (3)	
C19	0.46491 (14)	0.60771 (4)	0.42325 (13)	0.0152 (3)	
C14	0.89025 (14)	0.53788 (5)	0.64581 (13)	0.0166 (3)	
C7	0.81370 (14)	0.59140 (5)	0.47986 (13)	0.0163 (3)	
C29	0.21940 (15)	0.61670 (5)	−0.01674 (14)	0.0199 (3)	
H29	0.176380	0.606437	−0.104722	0.024*	
C30	0.29201 (15)	0.58490 (5)	0.07828 (13)	0.0182 (3)	
H30	0.298223	0.552962	0.057785	0.022*	
C24	0.53045 (14)	0.59869 (5)	0.55677 (13)	0.0169 (3)	
H24	0.552710	0.567797	0.588433	0.020*	
C20	0.42029 (14)	0.65335 (5)	0.37803 (13)	0.0157 (3)	
C26	0.34617 (14)	0.64906 (5)	0.23842 (13)	0.0158 (3)	
C33	0.46486 (15)	0.52786 (5)	0.32500 (13)	0.0173 (3)	
H33A	0.373802	0.510408	0.288448	0.021*	
H33B	0.506948	0.519086	0.418650	0.021*	
C36	0.72887 (15)	0.78399 (4)	0.61676 (13)	0.0160 (3)	
C39	0.73050 (16)	0.87537 (5)	0.51722 (13)	0.0188 (3)	
C10	0.85627 (14)	0.67016 (5)	0.63849 (13)	0.0173 (3)	
C13	0.83261 (14)	0.51464 (5)	0.52382 (13)	0.0171 (3)	
C27	0.26976 (15)	0.68025 (5)	0.14059 (13)	0.0183 (3)	
H27	0.260666	0.712053	0.160853	0.022*	
C11	0.80514 (14)	0.67383 (5)	0.50091 (14)	0.0181 (3)	
H11	0.785078	0.703834	0.462070	0.022*	
C41	0.85592 (15)	0.80421 (5)	0.60585 (13)	0.0187 (3)	
H41	0.942254	0.786587	0.630945	0.022*	
C9	0.89556 (14)	0.62684 (5)	0.69643 (13)	0.0169 (3)	
H9	0.935278	0.624193	0.789208	0.020*	
C22	0.53362 (15)	0.68257 (5)	0.59713 (13)	0.0175 (3)	
C8	0.87635 (14)	0.58719 (5)	0.61748 (13)	0.0164 (3)	
C21	0.45859 (15)	0.69087 (4)	0.46511 (13)	0.0168 (3)	
H21	0.433677	0.721754	0.434540	0.020*	
C15	0.94349 (15)	0.51210 (5)	0.76165 (14)	0.0187 (3)	
H15	0.982088	0.527344	0.843975	0.022*	
C40	0.85646 (15)	0.84895 (5)	0.55954 (13)	0.0191 (3)	
H40	0.943827	0.862017	0.556349	0.023*	
C23	0.56180 (14)	0.63634 (5)	0.64125 (13)	0.0175 (3)	
H23	0.604063	0.630782	0.732749	0.021*	
C18	0.82995 (15)	0.46586 (5)	0.51621 (14)	0.0201 (3)	
H18	0.792858	0.450233	0.434337	0.024*	
C38	0.60242 (15)	0.85476 (5)	0.51820 (13)	0.0195 (3)	
H38	0.515081	0.871706	0.485986	0.023*	
C17	0.88372 (15)	0.44135 (5)	0.63315 (15)	0.0221 (3)	
H17	0.882958	0.408274	0.630841	0.026*	
C32	0.56785 (15)	0.51229 (5)	0.25314 (14)	0.0189 (3)	
H32A	0.517105	0.515118	0.157582	0.023*	
H32B	0.589248	0.478860	0.272251	0.023*	
C16	0.93901 (15)	0.46396 (5)	0.75432 (14)	0.0212 (3)	
H16	0.973894	0.446068	0.832495	0.025*	
C28	0.20772 (15)	0.66384 (5)	0.01377 (14)	0.0203 (3)	
H28	0.156497	0.684726	−0.053655	0.024*	
C37	0.60095 (15)	0.81030 (5)	0.56511 (14)	0.0190 (3)	
H37	0.512048	0.796875	0.562854	0.023*	
C42	0.73111 (16)	0.92259 (5)	0.47377 (14)	0.0221 (3)	
C12	0.78308 (15)	0.63514 (5)	0.42023 (13)	0.0178 (3)	
H12	0.748271	0.638199	0.327350	0.021*	
C34	0.85826 (16)	0.71244 (5)	0.72398 (14)	0.0205 (3)	
H34A	0.868413	0.701739	0.813913	0.025*	
H34B	0.943460	0.731629	0.729302	0.025*	
C31	0.71121 (15)	0.53843 (5)	0.28535 (13)	0.0193 (3)	
H31A	0.776169	0.520158	0.250746	0.023*	
H31B	0.693366	0.568615	0.238387	0.023*	
C35	0.59918 (16)	0.72148 (5)	0.69368 (14)	0.0202 (3)	
H35A	0.526952	0.746430	0.683172	0.024*	
H35B	0.623737	0.709203	0.784210	0.024*	
C43	0.17529 (16)	0.66629 (5)	0.56184 (14)	0.0218 (3)	
H43A	0.094804	0.645051	0.557755	0.026*	
H43B	0.249688	0.648092	0.539858	0.026*	

### 10-(4-Cyanophenyl)-10,11,22,23-tetrahydro-9H,21H-5,8:15,12-bis(metheno)[1,5,11]triazacyclohexadecino[1,16-a:5,6-a']diindole dichloromethane monosolvate Atomic displacement parameters (Å^2^)

**Table:**

	*U*^11^	*U*^22^	*U*^33^	*U*^12^	*U*^13^	*U*^23^
Cl1	0.02377 (19)	0.0329 (2)	0.02753 (19)	0.00193 (14)	0.00544 (15)	0.00996 (14)
Cl2	0.0303 (2)	0.0375 (2)	0.01966 (18)	−0.00441 (15)	0.00875 (15)	−0.00085 (14)
N4	0.0151 (5)	0.0157 (5)	0.0147 (5)	−0.0003 (4)	0.0031 (4)	0.0019 (4)
N3	0.0157 (6)	0.0180 (5)	0.0174 (6)	−0.0003 (4)	0.0038 (5)	−0.0001 (4)
N5	0.0164 (6)	0.0164 (5)	0.0207 (6)	−0.0009 (4)	0.0035 (5)	0.0002 (4)
N6	0.0323 (8)	0.0246 (7)	0.0336 (7)	0.0020 (5)	0.0076 (6)	0.0075 (6)
C25	0.0118 (6)	0.0192 (6)	0.0170 (6)	−0.0011 (5)	0.0046 (5)	0.0032 (5)
C19	0.0123 (6)	0.0163 (6)	0.0178 (6)	−0.0020 (5)	0.0057 (5)	−0.0001 (5)
C14	0.0107 (6)	0.0178 (6)	0.0210 (7)	0.0000 (5)	0.0049 (5)	−0.0001 (5)
C7	0.0112 (6)	0.0191 (6)	0.0188 (6)	−0.0005 (5)	0.0050 (5)	−0.0001 (5)
C29	0.0144 (6)	0.0274 (7)	0.0167 (6)	−0.0012 (5)	0.0033 (5)	0.0006 (5)
C30	0.0151 (6)	0.0206 (6)	0.0183 (6)	−0.0017 (5)	0.0047 (5)	−0.0010 (5)
C24	0.0142 (6)	0.0178 (6)	0.0184 (6)	−0.0014 (5)	0.0049 (5)	0.0038 (5)
C20	0.0125 (6)	0.0179 (6)	0.0169 (6)	−0.0003 (5)	0.0052 (5)	0.0031 (5)
C26	0.0119 (6)	0.0190 (6)	0.0168 (6)	−0.0015 (5)	0.0048 (5)	0.0022 (5)
C33	0.0167 (6)	0.0154 (6)	0.0186 (6)	−0.0003 (5)	0.0043 (5)	0.0021 (5)
C36	0.0167 (6)	0.0167 (6)	0.0133 (6)	−0.0006 (5)	0.0033 (5)	−0.0031 (5)
C39	0.0226 (7)	0.0182 (6)	0.0147 (6)	0.0010 (5)	0.0049 (5)	−0.0008 (5)
C10	0.0120 (6)	0.0173 (6)	0.0212 (7)	−0.0017 (5)	0.0037 (5)	0.0006 (5)
C13	0.0126 (6)	0.0186 (6)	0.0200 (6)	0.0012 (5)	0.0052 (5)	0.0016 (5)
C27	0.0143 (6)	0.0193 (6)	0.0210 (7)	0.0007 (5)	0.0055 (5)	0.0044 (5)
C11	0.0134 (6)	0.0176 (6)	0.0222 (7)	−0.0002 (5)	0.0045 (5)	0.0041 (5)
C41	0.0150 (7)	0.0203 (7)	0.0197 (7)	0.0026 (5)	0.0043 (5)	0.0005 (5)
C9	0.0113 (6)	0.0201 (6)	0.0171 (6)	−0.0004 (5)	0.0016 (5)	0.0008 (5)
C22	0.0148 (6)	0.0199 (6)	0.0192 (6)	−0.0029 (5)	0.0075 (5)	−0.0001 (5)
C8	0.0099 (6)	0.0193 (6)	0.0188 (6)	−0.0001 (5)	0.0031 (5)	0.0022 (5)
C21	0.0150 (6)	0.0164 (6)	0.0198 (7)	−0.0010 (5)	0.0067 (5)	0.0020 (5)
C15	0.0135 (6)	0.0218 (7)	0.0197 (7)	0.0009 (5)	0.0038 (5)	0.0014 (5)
C40	0.0161 (7)	0.0220 (7)	0.0189 (6)	−0.0012 (5)	0.0055 (5)	−0.0001 (5)
C23	0.0141 (6)	0.0234 (7)	0.0147 (6)	−0.0027 (5)	0.0041 (5)	0.0018 (5)
C18	0.0164 (7)	0.0186 (7)	0.0244 (7)	0.0001 (5)	0.0054 (6)	−0.0020 (5)
C38	0.0170 (7)	0.0215 (7)	0.0185 (6)	0.0048 (5)	0.0040 (5)	−0.0014 (5)
C17	0.0178 (7)	0.0159 (6)	0.0317 (8)	0.0009 (5)	0.0070 (6)	0.0008 (6)
C32	0.0179 (7)	0.0190 (6)	0.0181 (6)	0.0001 (5)	0.0035 (5)	−0.0018 (5)
C16	0.0163 (7)	0.0217 (7)	0.0246 (7)	0.0024 (5)	0.0052 (6)	0.0068 (6)
C28	0.0150 (6)	0.0251 (7)	0.0190 (7)	0.0031 (5)	0.0032 (5)	0.0062 (5)
C37	0.0150 (6)	0.0218 (7)	0.0198 (7)	−0.0006 (5)	0.0052 (5)	−0.0022 (5)
C42	0.0219 (7)	0.0243 (7)	0.0182 (7)	0.0010 (6)	0.0040 (6)	0.0006 (6)
C12	0.0144 (6)	0.0217 (7)	0.0169 (6)	−0.0001 (5)	0.0042 (5)	0.0034 (5)
C34	0.0186 (7)	0.0171 (6)	0.0206 (7)	−0.0003 (5)	−0.0008 (6)	0.0006 (5)
C31	0.0190 (7)	0.0235 (7)	0.0153 (6)	−0.0003 (5)	0.0054 (5)	−0.0017 (5)
C35	0.0221 (7)	0.0210 (7)	0.0183 (6)	−0.0041 (5)	0.0078 (6)	−0.0007 (5)
C43	0.0206 (7)	0.0216 (7)	0.0232 (7)	−0.0015 (5)	0.0072 (6)	0.0014 (5)

### 10-(4-Cyanophenyl)-10,11,22,23-tetrahydro-9H,21H-5,8:15,12-bis(metheno)[1,5,11]triazacyclohexadecino[1,16-a:5,6-a']diindole dichloromethane monosolvate Geometric parameters (Å, º)

**Table:**

Cl1—C43	1.7693 (15)	C10—C34	1.5203 (19)
Cl2—C43	1.7664 (15)	C13—C18	1.4028 (19)
N4—C25	1.3840 (17)	C27—H27	0.9500
N4—C19	1.3821 (17)	C27—C28	1.385 (2)
N4—C33	1.4581 (17)	C11—H11	0.9500
N3—C7	1.3859 (17)	C11—C12	1.383 (2)
N3—C13	1.3869 (17)	C41—H41	0.9500
N3—C31	1.4594 (17)	C41—C40	1.3787 (19)
N5—C36	1.3731 (17)	C9—H9	0.9500
N5—C34	1.4664 (18)	C9—C8	1.3966 (19)
N5—C35	1.4759 (18)	C22—C21	1.3926 (19)
N6—C42	1.150 (2)	C22—C23	1.4070 (19)
C25—C30	1.3972 (19)	C22—C35	1.5193 (19)
C25—C26	1.4123 (19)	C21—H21	0.9500
C19—C24	1.3951 (19)	C15—H15	0.9500
C19—C20	1.4172 (18)	C15—C16	1.385 (2)
C14—C13	1.4170 (19)	C40—H40	0.9500
C14—C8	1.4454 (18)	C23—H23	0.9500
C14—C15	1.3975 (19)	C18—H18	0.9500
C7—C8	1.4128 (19)	C18—C17	1.388 (2)
C7—C12	1.3983 (19)	C38—H38	0.9500
C29—H29	0.9500	C38—C37	1.375 (2)
C29—C30	1.384 (2)	C17—H17	0.9500
C29—C28	1.407 (2)	C17—C16	1.399 (2)
C30—H30	0.9500	C32—H32A	0.9900
C24—H24	0.9500	C32—H32B	0.9900
C24—C23	1.3816 (19)	C32—C31	1.5357 (19)
C20—C26	1.4468 (18)	C16—H16	0.9500
C20—C21	1.3971 (19)	C28—H28	0.9500
C26—C27	1.4004 (19)	C37—H37	0.9500
C33—H33A	0.9900	C12—H12	0.9500
C33—H33B	0.9900	C34—H34A	0.9900
C33—C32	1.5307 (19)	C34—H34B	0.9900
C36—C41	1.4193 (19)	C31—H31A	0.9900
C36—C37	1.4178 (19)	C31—H31B	0.9900
C39—C40	1.398 (2)	C35—H35A	0.9900
C39—C38	1.397 (2)	C35—H35B	0.9900
C39—C42	1.435 (2)	C43—H43A	0.9900
C10—C11	1.4051 (19)	C43—H43B	0.9900
C10—C9	1.3889 (19)		
			
C25—N4—C33	126.04 (11)	C23—C22—C35	118.02 (12)
C19—N4—C25	108.37 (11)	C7—C8—C14	106.51 (12)
C19—N4—C33	125.51 (11)	C9—C8—C14	133.26 (12)
C7—N3—C13	108.45 (11)	C9—C8—C7	119.69 (12)
C7—N3—C31	124.50 (11)	C20—C21—H21	120.3
C13—N3—C31	127.02 (11)	C22—C21—C20	119.39 (12)
C36—N5—C34	122.81 (12)	C22—C21—H21	120.3
C36—N5—C35	122.37 (11)	C14—C15—H15	120.6
C34—N5—C35	114.81 (11)	C16—C15—C14	118.83 (13)
N4—C25—C30	128.48 (12)	C16—C15—H15	120.6
N4—C25—C26	109.19 (11)	C39—C40—H40	119.5
C30—C25—C26	122.30 (12)	C41—C40—C39	121.05 (13)
N4—C19—C24	129.57 (12)	C41—C40—H40	119.5
N4—C19—C20	109.55 (11)	C24—C23—C22	122.61 (12)
C24—C19—C20	120.88 (12)	C24—C23—H23	118.7
C13—C14—C8	106.50 (11)	C22—C23—H23	118.7
C15—C14—C13	119.90 (12)	C13—C18—H18	121.3
C15—C14—C8	133.59 (13)	C17—C18—C13	117.32 (13)
N3—C7—C8	109.36 (11)	C17—C18—H18	121.3
N3—C7—C12	129.63 (13)	C39—C38—H38	119.5
C12—C7—C8	120.91 (12)	C37—C38—C39	120.95 (13)
C30—C29—H29	119.2	C37—C38—H38	119.5
C30—C29—C28	121.61 (13)	C18—C17—H17	119.1
C28—C29—H29	119.2	C18—C17—C16	121.88 (13)
C25—C30—H30	121.5	C16—C17—H17	119.1
C29—C30—C25	117.06 (13)	C33—C32—H32A	108.0
C29—C30—H30	121.5	C33—C32—H32B	108.0
C19—C24—H24	121.2	C33—C32—C31	117.11 (11)
C23—C24—C19	117.55 (12)	H32A—C32—H32B	107.3
C23—C24—H24	121.2	C31—C32—H32A	108.0
C19—C20—C26	105.85 (11)	C31—C32—H32B	108.0
C21—C20—C19	119.80 (12)	C15—C16—C17	120.80 (13)
C21—C20—C26	134.23 (12)	C15—C16—H16	119.6
C25—C26—C20	106.80 (11)	C17—C16—H16	119.6
C27—C26—C25	119.31 (12)	C29—C28—H28	119.5
C27—C26—C20	133.84 (13)	C27—C28—C29	120.98 (13)
N4—C33—H33A	108.4	C27—C28—H28	119.5
N4—C33—H33B	108.4	C36—C37—H37	119.2
N4—C33—C32	115.60 (11)	C38—C37—C36	121.62 (13)
H33A—C33—H33B	107.4	C38—C37—H37	119.2
C32—C33—H33A	108.4	N6—C42—C39	178.31 (16)
C32—C33—H33B	108.4	C7—C12—H12	121.1
N5—C36—C41	122.10 (12)	C11—C12—C7	117.82 (12)
N5—C36—C37	121.52 (12)	C11—C12—H12	121.1
C37—C36—C41	116.38 (12)	N5—C34—C10	113.76 (11)
C40—C39—C42	121.43 (13)	N5—C34—H34A	108.8
C38—C39—C40	118.38 (12)	N5—C34—H34B	108.8
C38—C39—C42	120.19 (13)	C10—C34—H34A	108.8
C11—C10—C34	120.56 (12)	C10—C34—H34B	108.8
C9—C10—C11	119.51 (12)	H34A—C34—H34B	107.7
C9—C10—C34	119.77 (12)	N3—C31—C32	115.44 (11)
N3—C13—C14	109.14 (11)	N3—C31—H31A	108.4
N3—C13—C18	129.61 (13)	N3—C31—H31B	108.4
C18—C13—C14	121.25 (13)	C32—C31—H31A	108.4
C26—C27—H27	120.6	C32—C31—H31B	108.4
C28—C27—C26	118.72 (13)	H31A—C31—H31B	107.5
C28—C27—H27	120.6	N5—C35—C22	113.03 (11)
C10—C11—H11	119.0	N5—C35—H35A	109.0
C12—C11—C10	122.09 (12)	N5—C35—H35B	109.0
C12—C11—H11	119.0	C22—C35—H35A	109.0
C36—C41—H41	119.4	C22—C35—H35B	109.0
C40—C41—C36	121.28 (13)	H35A—C35—H35B	107.8
C40—C41—H41	119.4	Cl1—C43—H43A	109.4
C10—C9—H9	120.2	Cl1—C43—H43B	109.4
C10—C9—C8	119.60 (12)	Cl2—C43—Cl1	111.25 (8)
C8—C9—H9	120.2	Cl2—C43—H43A	109.4
C21—C22—C23	119.13 (12)	Cl2—C43—H43B	109.4
C21—C22—C35	122.58 (12)	H43A—C43—H43B	108.0
			
N4—C25—C30—C29	178.84 (13)	C10—C9—C8—C7	−1.7 (2)
N4—C25—C26—C20	−0.08 (14)	C13—N3—C7—C8	−2.19 (15)
N4—C25—C26—C27	−178.07 (12)	C13—N3—C7—C12	−178.57 (14)
N4—C19—C24—C23	174.81 (13)	C13—N3—C31—C32	60.02 (18)
N4—C19—C20—C26	4.64 (14)	C13—C14—C8—C7	−1.10 (14)
N4—C19—C20—C21	−172.03 (12)	C13—C14—C8—C9	170.10 (14)
N4—C33—C32—C31	52.46 (16)	C13—C14—C15—C16	0.2 (2)
N3—C7—C8—C14	2.03 (15)	C13—C18—C17—C16	−0.2 (2)
N3—C7—C8—C9	−170.61 (12)	C11—C10—C9—C8	−3.5 (2)
N3—C7—C12—C11	170.84 (13)	C11—C10—C34—N5	41.35 (18)
N3—C13—C18—C17	−178.94 (14)	C41—C36—C37—C38	−5.8 (2)
N5—C36—C41—C40	−172.75 (13)	C9—C10—C11—C12	4.5 (2)
N5—C36—C37—C38	173.22 (12)	C9—C10—C34—N5	−134.01 (13)
C25—N4—C19—C24	174.70 (13)	C8—C14—C13—N3	−0.20 (15)
C25—N4—C19—C20	−4.79 (14)	C8—C14—C13—C18	179.77 (12)
C25—N4—C33—C32	66.61 (17)	C8—C14—C15—C16	179.01 (14)
C25—C26—C27—C28	−1.04 (19)	C8—C7—C12—C11	−5.2 (2)
C19—N4—C25—C30	−175.24 (13)	C21—C20—C26—C25	173.22 (14)
C19—N4—C25—C26	2.97 (14)	C21—C20—C26—C27	−9.2 (3)
C19—N4—C33—C32	−116.79 (14)	C21—C22—C23—C24	6.5 (2)
C19—C24—C23—C22	−1.7 (2)	C21—C22—C35—N5	−73.73 (17)
C19—C20—C26—C25	−2.74 (14)	C15—C14—C13—N3	178.90 (12)
C19—C20—C26—C27	174.83 (14)	C15—C14—C13—C18	−1.1 (2)
C19—C20—C21—C22	−3.50 (19)	C15—C14—C8—C7	179.97 (14)
C14—C13—C18—C17	1.1 (2)	C15—C14—C8—C9	−8.8 (3)
C14—C15—C16—C17	0.7 (2)	C40—C39—C38—C37	2.9 (2)
C7—N3—C13—C14	1.47 (15)	C23—C22—C21—C20	−3.69 (19)
C7—N3—C13—C18	−178.50 (14)	C23—C22—C35—N5	100.31 (14)
C7—N3—C31—C32	−117.55 (14)	C18—C17—C16—C15	−0.7 (2)
C30—C25—C26—C20	178.27 (12)	C38—C39—C40—C41	−2.4 (2)
C30—C25—C26—C27	0.3 (2)	C28—C29—C30—C25	−1.2 (2)
C30—C29—C28—C27	0.5 (2)	C37—C36—C41—C40	6.31 (19)
C24—C19—C20—C26	−174.91 (12)	C42—C39—C40—C41	177.50 (13)
C24—C19—C20—C21	8.43 (19)	C42—C39—C38—C37	−177.01 (13)
C20—C19—C24—C23	−5.75 (19)	C12—C7—C8—C14	178.78 (12)
C20—C26—C27—C28	−178.37 (14)	C12—C7—C8—C9	6.1 (2)
C26—C25—C30—C29	0.8 (2)	C34—N5—C36—C41	−4.27 (19)
C26—C20—C21—C22	−179.02 (14)	C34—N5—C36—C37	176.72 (12)
C26—C27—C28—C29	0.7 (2)	C34—N5—C35—C22	−75.00 (15)
C33—N4—C25—C30	1.8 (2)	C34—C10—C11—C12	−170.87 (13)
C33—N4—C25—C26	−179.94 (12)	C34—C10—C9—C8	171.90 (12)
C33—N4—C19—C24	−2.4 (2)	C31—N3—C7—C8	175.76 (12)
C33—N4—C19—C20	178.11 (12)	C31—N3—C7—C12	−0.6 (2)
C33—C32—C31—N3	43.94 (17)	C31—N3—C13—C14	−176.42 (12)
C36—N5—C34—C10	−106.00 (14)	C31—N3—C13—C18	3.6 (2)
C36—N5—C35—C22	104.09 (15)	C35—N5—C36—C41	176.71 (12)
C36—C41—C40—C39	−2.3 (2)	C35—N5—C36—C37	−2.29 (19)
C39—C38—C37—C36	1.4 (2)	C35—N5—C34—C10	73.08 (15)
C10—C11—C12—C7	−0.1 (2)	C35—C22—C21—C20	170.28 (12)
C10—C9—C8—C14	−171.95 (14)	C35—C22—C23—C24	−167.77 (13)

### 10-(4-Cyanophenyl)-10,11,22,23-tetrahydro-9H,21H-5,8:15,12-bis(metheno)[1,5,11]triazacyclohexadecino[1,16-a:5,6-a']diindole dichloromethane monosolvate Hydrogen-bond geometry (Å, º)

*Cg*1, *Cg*2, *Cg*3 and *Cg*4 are the centroids of rings C36–C41, C13–C18, C7–C12 and C19–C24, respectively.

**Table:**

*D*—H···*A*	*D*—H	H···*A*	*D*···*A*	*D*—H···*A*
C12—H12···*Cg*1^i^	0.95	2.93	3.8374 (15)	159
C33—H33*A*···*Cg*2^ii^	0.99	2.91	3.6402 (17)	131
C43—H43*A*···*Cg*3^iii^	0.99	2.56	3.4632 (17)	151
C43—H43*B*···*Cg*4	0.99	2.54	3.4277 (17)	149

Symmetry codes: (i) *x*, −*y*+1/2, *z*−3/2; (ii) −*x*+1, −*y*+1, −*z*+1; (iii) *x*−1, *y*, *z*.
